# Physiological mechanisms and therapeutic targets in chronic cough

**DOI:** 10.14814/phy2.71036

**Published:** 2026-08-06

**Authors:** James C. Brown, Katherine L. Rhatigan, Oliver J. Price, Peter S. P. Cho

**Affiliations:** ^1^ School of Biomedical Sciences, Faculty of Biological Sciences University of Leeds Leeds UK; ^2^ Department of Respiratory Medicine King's College Hospital NHS Foundation Trust London UK; ^3^ Centre for Human and Applied Physiological Sciences King's College London London UK; ^4^ Department of Respiratory Medicine Leeds Teaching Hospitals NHS Trust Leeds UK

**Keywords:** airway sensory nerves, chronic cough, cough hypersensitivity, therapeutic targets, vagal afferent pathways

## Abstract

Cough is a vital defensive reflex that safeguards the airways; however, it can become maladaptive and pathological. Chronic cough (>8 weeks in duration) affects up to 10% of the global adult population and is associated with considerable health status impairment. The understanding of cough physiology remains limited. Cough reflex hypersensitivity is conventionally considered to underpin the pathophysiology of chronic cough, primarily mediated by peripheral nerve dysfunction. Whilst this framework has led to important mechanistic insights and effective pharmacotherapies, recent functional neuroimaging studies have demonstrated equally important central mechanisms. Taken together, cough reflex hypersensitivity may not be mediated exclusively by peripheral nerve dysfunction, and the answer may lie within a complex interplay of central and peripheral neural physiology. In the pathophysiology of chronic cough, impaired voluntary suppression also appears to be important. This review will provide an overview of the physiology and pathophysiology of chronic cough and attempts in pharmacotherapy to date.

## 1 INTRODUCTION

Cough is a defensive respiratory manoeuvre that facilitates the clearance of inhaled irritants, pathogens and superfluous secretions (Morice, Dicpinigaitis, et al., 2021). However, when persistent or excessive, cough becomes maladaptive and pathological (Bali et al., 2024; Kubo et al., 2021; McGarvey et al., 2023). Clinically, pathological cough is defined by its duration: acute (<3 weeks), subacute (3–8 weeks) and chronic (>8 weeks) (Morice, Dicpinigaitis, et al., 2021). Acute and subacute cough are typically infective or inflammatory and are often self‐limiting, whereas chronic cough represents a more complex, burdensome condition.

Chronic cough affects approximately 10% of the global adult population and is termed refractory chronic cough (RCC) when it persists despite exhaustive investigation and optimal treatment (Zhang & Morice, 2024). RCC is associated with a wide range of physical and psychosocial co‐morbidities, including syncope, chest pain, lethargy, depression and anxiety (Cho et al., 2024; Drake et al., 2023; French et al., 2017; Hirons et al., 2022; McGarvey et al., 2006; Morice, Dicpinigaitis, et al., 2021; Song et al., 2015). Chronic cough also carries a substantial economic burden, with a reported mean (SD) annual healthcare cost of approximately £1663 (£747) per person (Cho et al., 2022). Although chronic cough has long been viewed as a symptom of chronic respiratory conditions, such as asthma and COPD, it is now increasingly recognised as a distinct clinical entity (Zhang & Morice, 2024).

RCC is not associated with any respiratory pathology and is increasingly believed to be secondary to neural dysfunction. RCC was associated with cough reflex hypersensitivity to tussive stimuli in early experimental and clinical reports (Kim et al., 2024; Singh et al., 2020). These observations led to the hypothesis that RCC is a neuropathic disorder mediated by cough reflex hypersensitivity (Zhang & Morice, 2024). However, cough is not solely a reflexive process; it can also be voluntarily initiated or suppressed. Indeed, recent functional neural imaging and physiological studies suggest that impaired central control diminished volitional suppression, which may contribute to the persistence of cough in RCC (Ando et al., 2016; Cho et al., 2019). These findings suggest that both peripheral sensory dysfunction and central neural dysregulation are important in its pathophysiology.

Despite these advances, the relative contributions of peripheral and central mechanisms in RCC remain incompletely defined, and this uncertainty continues to limit the development of effective, targeted therapies. A clearer understanding of these neuromechanisms is critical for developing targeted antitussive pharmacotherapies. This review aims to present our current understanding of the physiology and pathophysiology of chronic cough, with the latter being presented through RCC. We will also discuss attempts in pharmacotherapies guided by neural physiology to date, the neural mechanisms governing RCC and to critically evaluate the emerging pharmacological approaches for its management.

## 2 PHYSIOLOGY OF COUGH

**FIGURE 1:**
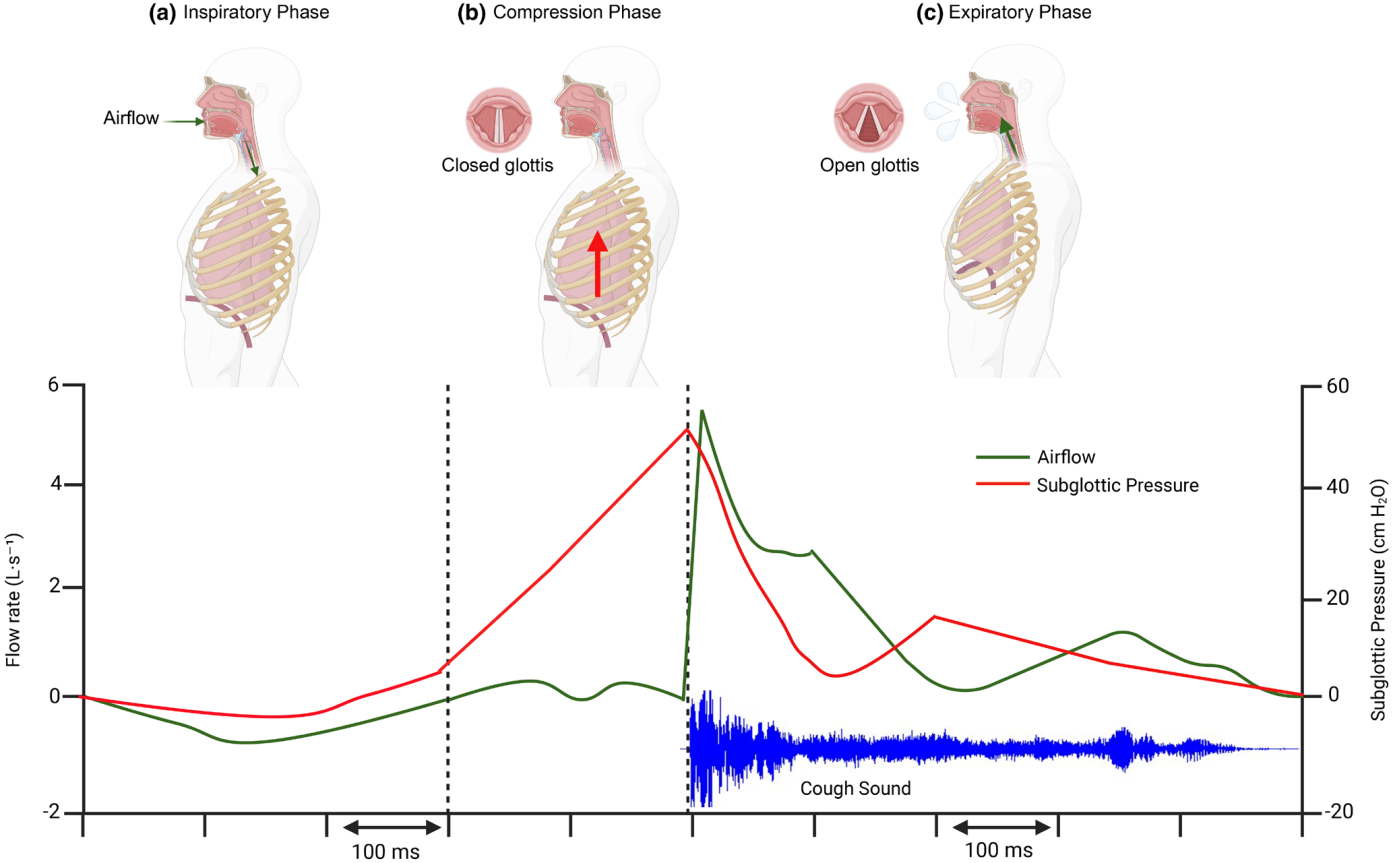
Mechanics of cough. (a) Initial inspiratory phase: Involving a variable inhalation of up to 50% of vital capacity, increasing intrathoracic volume and optimizing expiratory muscle length‐tension properties. (b) Compression phase: Brief closure of the glottis (~ 200 ms) and contraction of the expiratory muscles increase intrathoracic pressure (up to 300 mmHg). (c) Expiratory phase: The glottis opens, producing a rapid supramaximal airflow (30–50 ms; up to 12 L·s^−1^), followed by a longer, lower flow (200–500 ms; 3–4 L·s^−1^). This high‐velocity airflow clears airway debris, whilst enhanced lung movement and mucociliary transport aid peripheral clearance. Created in BioRender. Brown, J. (2026) https://biorender.com/809ysd8.

The cough motor pattern is well characterised as a coordinated respiratory motor act that comprises sequential inspiratory, compressive and expiratory phases that generate high intrathoracic pressures and rapid expiratory airflow to effectively clear the airways (Figure 1). The initiation and regulation of cough are underpinned by complex neurophysiological processes that can broadly be categorised into peripheral and central processes. Dysregulation of these pathways is thought to play a key role in the pathogenesis of RCC. Therefore, the following sections will explore the peripheral and central mechanisms involved in cough, how these processes are altered by RCC and emerging pharmacotherapies targeting these pathways.

## 3 PERIPHERAL NEUROPHYSIOLOGY

Peripheral mechanisms underlying chronic cough are characterised by heightened sensitivity of airway sensory afferents to a range of chemical and mechanical stimuli (Cho et al., 2019; Fowles et al., 2017; Millqvist & Löwhagen, 1996; Moe et al., 2024). The lungs and airways are innervated by a dense network of peripheral sensory nerve fibres comprised of a (Shen et al., 2022) plethora of different afferent receptors in the respiratory system that collectively contribute to cough regulation, rather than in isolation (Drake et al., 2023). Indeed, Belvisi and colleagues demonstrated that responses to tussive agents were different across chronic respiratory diseases, thus seeding the idea of different phenotypes and endotypes of cough in different diseases (Belvisi et al., 2016). A comprehensive overview of all receptors involved in cough is beyond the scope of this article; therefore, this section will focus on those most strongly implicated in cough hypersensitivity and current therapeutic development.

### 3.1 Vagal afferents

As with any reflex arc, the initial phase of cough involves the encoding of action potentials by afferent nerves in response to a tussive stimulus and the reconfiguration of brainstem respiratory motor drive (Shen et al., 2022). Vagal afferents appear to have a key role in initiating the cough reflex, as bilateral vagotomy significantly abates the cough reflex in both animals (Canning et al., 2004; Shen et al., 2021) and humans (Zhang, Ge, et al., 2024). Vagal sensory afferents originate from two distinct sites, the nodose (inferior) and jugular ganglia (superior), which are situated bilaterally at the skull base (Nassenstein et al., 2010). These nodose and jugular afferent neurons have a peripheral axon that terminates in the airways or lung parenchyma and a central axon that terminates in the brainstem, enabling the sensory input to be processed and integrated into respiratory control (Nassenstein et al., 2010).

**FIGURE 2:**
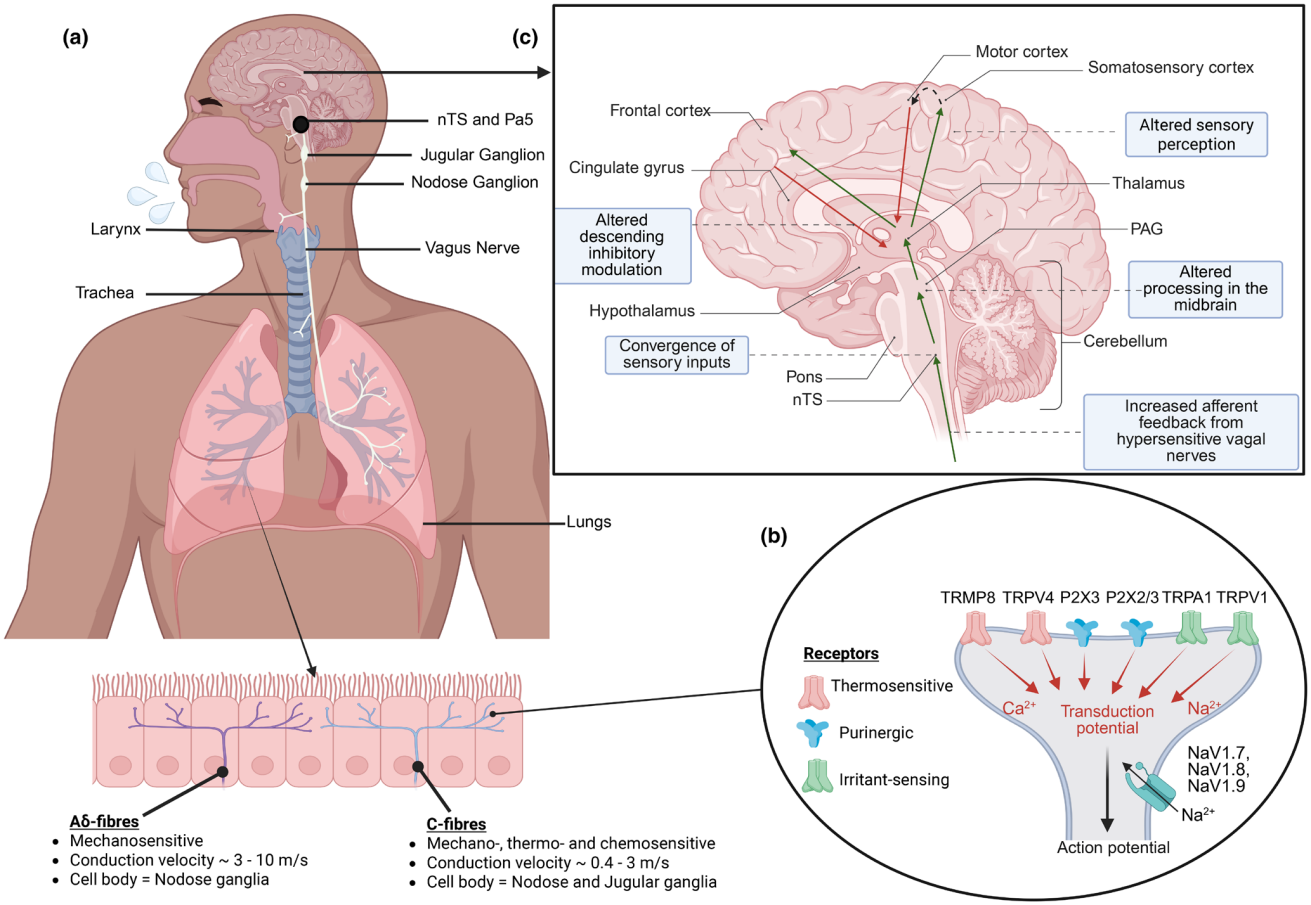
Current understanding of the central and peripheral mechanisms underlying chronic cough. (a) Airway sensory nerves originating in the nodose and jugular ganglia (Aδ and C‐fibres) are activated by mechanical and chemical stimuli. These nerve signals from the periphery are relayed to the nucleus of the solitary tract and the paratrigeminal nucleus in the medulla oblongata, where they ascend to a distributed central network that supports cough. (b) Current receptor channels thought to be involved in the cough reflex. Activation of these receptors and channels leads to ionic influx into the nerve terminal and the generation of transduction potentials. Above a certain threshold, voltage‐gated sodium channels (NaVs) open, propagating an action potential centrally to evoke cough. (c) The ascending and descending central pathways involved in cough and the proposed altered central processing in people with chronic cough. Created in BioRender. Brown, J. (2026) https://biorender.com/5oeaddf.

The transduction of a stimulus into electrochemical activity in the nerve terminals of cough sensory nerves occurs when G‐protein‐coupled receptors (GCPRs) and ion channels are activated (Al‐Kandery et al., 2021; Gao et al., 2021; Grace et al., 2012; Hewitt et al., 2016). Peripheral activation of individual GCPRs is stimulus‐dependent and will be discussed individually in due course. Activation of these receptors and ion channels leads to an ionic influx into the nerve terminal and the initiation of a transduction potential (Al‐Kandery et al., 2021; Grace et al., 2012; Kim et al., 2023; Sun et al., 2020). If the localised transduction current was of sufficient magnitude, the terminal voltage‐gated sodium channels open and propagate action potential waves centrally, which evokes cough (Al‐Kandery et al., 2021; Grace et al., 2012; Guilleminault et al., 2024; Kim et al., 2023; Sun et al., 2020) (Figure 2). In individuals with RCC, the conventional belief was cough reflex hypersensitivity, which may be manifested as an increase in sensory nerve excitability via upregulation of receptor ion channels and the concomitant activation of these channels by inflammatory mediators (Grace et al., 2012; Gruenberg et al., 2004). These mechanisms include both ionotropic receptors, such as P2X3, which directly mediate rapid depolarisation of sensory nerves in response to extracellular ATP (Li et al., 2019; North, 2002), and metabotropic receptors that signal via intracellular pathways to modulate excitability (Mazzone & Undem, 2016; Sun et al., 2020). However, emerging evidence suggests that structural alterations within the airway sensory network may also contribute to cough hypersensitivity (Shapiro et al., 2021). Recently, Shapiro and colleagues reported increased airway epithelial sensory nerve length and density in patients with RCC compared to healthy individuals (Shapiro et al., 2021). Meanwhile, substance P expression was not significantly different between RCC and healthy individuals. Together, these findings suggest that cough hypersensitivity is due to both functional sensitisation and structural remodelling of the sensory nerves. Thus, an understanding of the distinct afferent subtypes and how structural and functional alterations affect the ionotropic and metabotropic signalling pathways is critical in elucidating the mechanisms underpinning cough hypersensitivity and will be discussed in the subsequent sections (Davies et al., 1987; West et al., 2015; Widdicombe, 2003).

#### 3.1.1 A‐fiber receptors

Vagal afferents with action potential velocities in the “A” range primarily reside in the nodose ganglia (Hunter & Undem, 1999). These myelinated afferents are generally sensitive to mechanical stimuli and include receptors that are important for the physiological control of respiration, such as slowly adapting receptors (SARs) and rapidly adapting receptors (RARs) involved in the Hering–Breuer inflation and deflation reflexes (Davies et al., 1987; Widdicombe, 2003).

Typically, A‐fibres are subclassified into Aβ‐fibres (fastest conduction velocity) and Aδ‐fibres (intermediate conduction velocity) (Mazzone & Undem, 2016). Aβ‐fibres are not considered to be part of the cough‐reflex arc. In contrast, a specialised subset of rapidly adapting Aδ‐fibres responds to noxious mechanical and acidic stimuli, such as inhaled particulate matter, mucus accumulation and the aspiration of food/gastrointestinal contents (Canning et al., 2004). These Aδ‐fibres are sometimes referred to as the “cough receptors”. The Aδ‐fibres associated with the cough reflex in guinea pigs terminate almost exclusively in the larynx, trachea and bronchi, and are sensitive to mechanical and acidic stimulations, even under anaesthesia (Canning et al., 2004). More recently, evidence has emerged that high quantities of similar structures are found in human airways (West et al., 2015), whilst mice and rats, which have a distinctly less sensitive cough reflex, have a relative paucity of these fibres (Mazzone et al., 2009). These cough receptors are thought to have a selective advantage by reducing the potentially lethal complications of aspiration (Mazzone et al., 2009). Despite having a crucial role in cough initiation, the specific role of Aδ‐fibres in RCC remains incompletely defined. Current evidence suggests that inflammatory mediators appear to preferentially sensitise C‐fibres that are enriched with ionotropic and metabotropic receptors, such as P2X3 and TRPV1 (discussed in subsequent sections), with these fibres making up most (~70%) airway vagal afferents (Birrell et al., 2009; Birring et al., 2023; Bonvini et al., 2016; Grace et al., 2012; Lee et al., 2003; Morice et al., 2019). This does not exclude a role for Aδ‐fibres; rather, their contribution may arise through increased recruitment secondary to structural airway remodelling and increased central amplification of afferent input (Shapiro et al., 2021). It's therefore possible that the role of the Aδ‐fibres in RCC is to translate the heightened sensory signals into the motor act of coughing.

#### 3.1.2 C‐fibre receptors

Unmyelinated C‐fibres are the most common afferent fibres in the respiratory system and have traditionally been categorised as bronchial and pulmonary fibres based on their respective arterial supplies (Undem & Nassenstein, 2009). C‐fibres can alternatively be categorised according to their ganglionic origins into jugular or nodose fibres, each with its own differential protein expression and function (Mazzone & Undem, 2016). Anatomically, extrapulmonary C‐fibres are largely jugular in origin, whilst both jugular and nodose fibres contribute to intrapulmonary innervations (Mazzone & Undem, 2016). C‐fibres express numerous ligand‐gated ion channels, which act as polymodal nociceptors. Ionotropic receptors, expressed preferentially on C‐fibres, including transient receptor potential channels TRPV1 and TRPA1, play a key role in cough triggered by inhaled irritants such as capsaicin, citric acid and bradykinin in both awake humans and guinea pigs (Cho et al., 2019; Chou et al., 2018; Zhang et al., 2018). In human neuronal cells, inflammatory processes can upregulate TRP receptors on C‐fibres, and bronchial biopsies from patients with chronic cough similarly demonstrate increased TRP receptor expression (Abdullah et al., 2014; Gruenberg et al., 2004).

The relationship between C‐fibre activation and cough is, however, complex. Indeed, evidence suggests opposing roles for jugular and nodose C‐fibre subtypes (Chou et al., 2018). In guinea pigs, citric acid and capsaicin evoke cough, with TRPV1 and TRPA1 receptors more abundant on jugular fibres, implicating them in cough sensitisation (Chou et al., 2018). Conversely, nodose‐selective agonists (adenosine, 2‐methyl‐5‐HT) suppress citric acid‐ and capsaicin‐induced cough (Chou et al., 2018). These findings indicate that stimuli favouring jugular over nodose activation may drive excessive coughing. Whilst selective activation of nodose fibres is unlikely to be therapeutic due to associated dyspnoea, future strategies may focus on inhibiting jugular C‐fibre activity through receptor‐specific antagonism.

Ionotropic and metabotropic receptors are expressed in vast quantities on C‐fibres. In individuals with RCC, these receptors demonstrate heightened sensitivity to inhaled tussive agonists, including capsaicin (via TRPV1) and ATP (via P2X3), compared with healthy controls (Figure 2). Notably, several of the most promising emerging antitussive therapies target these receptors. Therefore, the following sections focus on receptors preferentially expressed on C‐fibres that have been implicated in RCC pathophysiology and evaluate their potential clinical utility.

#### 3.1.3 P2X purinoceptor 3

Growing evidence indicates a mechanistic role for the purinergic P2X3 and P2X/3 receptor in the activation of airway sensory fibres that drive the cough reflex (Birring, Cardozo, et al., 2024; Guilleminault et al., 2024; McGarvey et al., 2022; McGarvey, Sher, et al., 2023; McGarvey, Smith, et al., 2023; Morice, Smith, et al., 2021; Niimi et al., 2022). Using ex vivo isolated, perfused lung‐nerve preparations from guinea pigs, increased action potential discharge from nodose C‐fibres has been demonstrated following exposure to histamine and methacholine (Weigand et al., 2012). P2X3‐containing receptors are activated by adenosine 5′ triphosphate (ATP), which is released extracellularly by injured or activated epithelial cells (Dosch et al., 2018). Indeed, inhaled ATP induces cough in a dose‐dependent manner in both healthy individuals and patients with RCC. In addition, the concentration of ATP required to elicit cough was lower in patients with RCC compared to healthy individuals, thus suggesting cough hypersensitivity involving the P2X3 pathway. Whilst the latter demonstrated features of cough reflex hypersensitivity with ATP‐evoked cough (Fowles et al., 2017; Moe et al., 2024). Although the precise mechanistic pathways remain incompletely understood, respiratory conditions associated with RCC and airway inflammation are characterised by elevated extracellular ATP and heightened sensory nerve responsiveness (Basoglu et al., 2005; Moe et al., 2024). The release of ATP into the airway presumably stimulates vagal sensory neurons by binding to purinergic receptors, including P2X3 (Muccino & Green, 2019). Summarily, pharmacological antagonism of P2X3 and P2X/3 receptors attenuated tussive response in humans (Weigand et al., 2012).

Recently, P2X3 receptor antagonists have been investigated extensively in the treatment of RCC (Birring et al., 2023; Birring, Cardozo, et al., 2024; Friedrich et al., 2023; McGarvey et al., 2022; McGarvey, Sher, et al., 2023; McGarvey, Smith, et al., 2023; Morice et al., 2019; Morice, Smith, et al., 2021; Niimi et al., 2022). Notably, Gefapixant is the first in‐class efficacious P2X2/3 antagonist within phase 3 studies (Birring et al., 2023; Birring, Cardozo, et al., 2024; McGarvey et al., 2022; McGarvey, Sher, et al., 2023; Morice et al., 2019). Compared to placebo, Gefapixant showed a significant reduction in 24‐h objective cough frequency (18.5%) and was more likely to improve cough‐specific health status, Leicester Cough Questionnaire (LCQ) score, beyond the minimally clinically important threshold of 1.3 (OR 1.41; both *p* < 0.05) (McGarvey et al., 2022). This suggests that the observed reduction in cough frequency in Gefapixant was more likely to improve the physical, psychological and social well‐being of patients than control.

Although Gefapixant was approved by the European Medicines Agency and is now available in several countries, including Switzerland and Japan, regulatory agencies, including the Food and Drug Administration (FDA) and National Institute for Health and Care Excellence (NICE), expressed concerns regarding the clinical meaningfulness of the treatment effect and high incidence of dysgeusia (~50%), which led to notable rates of discontinuation (McGarvey et al., 2022; Birring, Cardozo, et al., 2024; McGarvey, Sher, et al., 2023; European Medicines Agency (EMA), n.d.; National Institute for Health and Care Excellence (NICE), n.d.). Other P2X3 antagonists are currently being investigated, with some (Sivopixant and Camlipixant) demonstrating improved tolerability but more variable efficacy. In contrast to many chronic respiratory diseases, RCC is not associated with increased mortality or hospitalisation, highlighting the complexity of assessing the cost‐effectiveness of antitussive therapies (Dicpinigaitis et al., 2023; Friedrich et al., 2023; McGarvey, Smith, et al., 2023; Morice, Smith, et al., 2021; Niimi et al., 2022; Smith, Birring, et al., 2025; Smith, Morice, et al., 2025).

#### 3.1.4 Transient receptor potential cation channel

Transient receptor potential vanilloid 1 (TRPV1) channels are non‐selective, Ca^2+^‐preferential cation channels primarily expressed on C‐fibres and, to a lesser extent, Aδ‐fibres (Adcock, 2009; Alawi & Keeble, 2010). Activated by ligands such as capsaicin and citric acid, these channels mediate potent tussive responses, and aerosolised forms of these agents are widely used in cough challenge testing (Cho et al., 2019; Chou et al., 2018; Holt et al., 2023; King et al., 2021). In RCC, TRPV1 expression is increased 4.4‐fold compared to health, and patients exhibit heightened capsaicin sensitivity, prompting interest in TRPV1 as a therapeutic target (Cho et al., 2019; Gruenberg et al., 2004; Mitchell et al., 2005; Moe et al., 2024). Unfortunately, recent studies have shown limited clinical benefit. In a placebo‐controlled crossover study, SB‐705498 (600 mg) raised the five‐cough threshold but failed to reduce spontaneous cough or improve symptoms (Khalid et al., 2014). Similarly, XEN‐D0501, a TRPV1 antagonist approximately 1000‐fold more potent than SB‐705498, increased cough thresholds over 14 days yet did not affect cough frequency or patient‐reported outcomes (Belvisi et al., 2017). Thus, whilst TRPV1 antagonists blunt capsaicin‐induced cough, they do not appear to alleviate spontaneous cough, challenging the utility of capsaicin challenge testing and suggesting that single‐channel TRPV1 blockade is unlikely to provide meaningful clinical benefit.

TRPV4, like TRPV1, responds to pH, temperature and mechanical stress but is mainly expressed in non‐neuronal airway cells, where it promotes bronchoconstriction (Baxter et al., 2014; Jentsch Matias de Oliveira et al., 2020; Toft‐Bertelsen et al., 2017). Hypo‐osmolar solutions activate Aδ‐fibres via TRPV4‐driven ATP release and P2X3 signalling, provoking cough in guinea pigs and depolarisation in human vagal nerves (Bonvini et al., 2016). Although P2X3 antagonists block these responses, TRPV4 antagonists have failed clinically; the only placebo‐controlled trial of GSK2798745 in RCC was terminated early after a 32% increase in cough frequency after 7 days (Ludbrook et al., 2019). The transient receptor potential ankyrin 1 (TRPA1) channel is widely expressed in C‐fibres of the respiratory tract, often co‐localised with TRPV1 (Brozmanova et al., 2012). These receptors are susceptible to reactive ligands found in cigarette smoke and air pollutants, including acrolein, chlorine and crotonaldehyde (Brozmanova et al., 2012). TRPA1 is also activated by cold air and numerous endogenous inflammatory mediators (Dong et al., 2020; Steib et al., 2025). There is also compelling evidence that TRPA1 is a key mediator of cough responses to environmental irritants (Birrell et al., 2009). Using an integrated approach encompassing molecular, ex vivo, in vivo, and human studies, it was demonstrated that TRPA1 agonists such as cinnamaldehyde depolarise both guinea pig and human vagal nerves and reliably evoke cough in guinea pigs and healthy volunteers (Birrell et al., 2009). Although the TRPA1 antagonist GRC17536 inhibited citric acid–induced Ca^2+^ influx in TRPA1‐expressing cells in vitro and suppressed the cough reflex in awake guinea pigs (Mukhopadhyay et al., 2014), a Phase 2a clinical trial failed to show clinical benefit in patients with RCC (Morice, 2017).

Finally, the transient receptor potential melastatin 8 (TRPM8) has recently shown promise as an anti‐tussive target. This receptor is primarily expressed in neurons and is activated by cool temperatures and chemical compounds that have a ‘cooling’ effect, such as menthol and eucalyptus (Mergler et al., 2013; Peier et al., 2002; Stinson et al., 2023). In healthy volunteers, 30 mg of menthol, administered immediately before a capsaicin challenge, increased the 5‐cough threshold from 7.81 μM to 15.63 μM (Katayama et al., 2025). Furthermore, in a proof‐of‐concept trial, two 40 mg doses of orally administered AX‐8 (a TRPM8 agonist) reduced cough frequency in patients with RCC compared to placebo treatment, supporting TRPM8 activation as a novel therapeutic approach (Smith et al., 2023). Building on these encouraging findings, AX‐8 warrants evaluation in large, multicentre, randomised, double‐blind trials to confirm long‐term efficacy and safety and establish optimal dosing regimens.

Overall, multiple peripheral receptors elicit a tussive response upon provocation in a dose–response manner, both in vitro and in vivo. These responses support a potential mechanistic role; however, pharmacological antagonism of these receptors has yielded mixed results. Taken together, physiological interrogation of peripheral neuropathways in RCC may provide novel insight, although caution is warranted in interpreting mechanistic and therapeutic implications pending more comprehensive evaluation. Notably, these approaches have already yielded promising translational benefits. Although peripheral sensitisation of airway afferent nerves is a key driver of RCC, it does not fully account for the heightened cough reflex observed in many patients. Sensory inputs arising from the airways are relayed via vagal afferents to the brainstem and higher cortical areas (Moe et al., 2024). These signals are integrated and modulated in these central areas and ultimately lead to conscious perception as the urge‐to‐cough (Moe et al., 2024; Moe et al., 2026). Evidence increasingly suggests that alterations in these central networks contribute to cough hypersensitivity, highlighting that RCC should be considered as a disorder of both peripheral and central neural processing (Moe et al., 2024; Moe et al., 2026). Accordingly, the following section focuses on the central pathways that govern cough generation and modulation.

## 4 CENTRAL PATHWAY

The central mechanisms responsible for generating cough are influenced by cough type. In humans, cough can be reflexive, voluntary or evoked, the latter of which is preceded by an urge to cough sensation (Hegland et al., 2012; Mazzone et al., 2011). Cough can also be suppressed in response to a tussigenic stimulus (Mazzone et al., 2011). Although our current understanding remains incomplete, animal studies (Bongianni et al., 2005; Flor et al., 2019; Poliacek et al., 2017; Simera et al., 2024) and the use of brain imaging technology, such as functional magnetic resonance imaging (fMRI) (Mazzone et al., 2011; Moe et al., 2024; Moe et al., 2026) and positron emission tomography (PET) (Sugi et al., 2024) in human studies, have allowed for these central pathways to be somewhat elucidated and for distinctions to be made between cough types.

### 4.1 Role of the brainstem and the midbrain

Glutamatergic vagal afferents (Aδ and C fibres) terminate in the caudal nucleus tractus solitarius (nTS), the primary sensory hub for cough initiation (Hass & Benarroch, 2024). Recent evidence identifies Tac1‐expressing nTS neurons as central to cough pattern generation (Gannot et al., 2024). Using single‐cell transcriptomics, viral circuit tracing, optogenetics and chemogenetics in mice, these neurons integrated vagal input and efferently projected to medullary circuits that control glottic closure (nucleus ambiguus) and expiratory drive (caudal ventral respiratory group) (Gannot et al., 2024). Optogenetic activation evoked cough‐like responses, whereas chemogenetic silencing or ablation inhibited responses to tussive stimuli without affecting baseline respiration (Gannot et al., 2024). The nTS also communicates with pontomedullary respiratory groups, including the Kölliker‐Fuse nucleus, Bötzinger (BötC) and preBötzinger (preBötC) complexes and rostral/caudal ventral respiratory groups, implicated in rhythmogenesis (Gannot et al., 2024; Hass & Benarroch, 2024). Experimental data in anaesthetised cats indicate that excitatory stimulation of glutamatergic receptors in the preBötC enhances cough intensity, whereas kynurenic acid microinjection attenuates cough magnitude without abolishing the pattern, implicating additional sites such as the nTS in rhythmogenesis (Shen et al., 2022). The BötC and rVRG contribute to the inspiratory and expiratory phases (Cinelli et al., 2020; Gannot et al., 2024), whilst selective blockade in the cVRG with kynurenic acid suppresses both components in rabbits, suggesting its role in generating the full cough motor sequence (Bongianni et al., 2005; Cinelli et al., 2020). Cerebellar involvement is supported by reduced cough frequency after cerebellectomy in cats (Xu et al., 1997) and cerebellar activation during voluntary and reflex cough in humans (Sugi et al., 2024), highlighting distributed brainstem–cerebellar networks in cough control.

As might be expected given the rather limited understanding of the central neuropathophysiology in RCC, CNS‐targeted pharmacotherapy development has been relatively sparse compared to peripherally targeted counterparts. Of note, preclinical studies indicate that tachykinins, particularly Substance P, are released by vagal C‐fibres both peripherally in the airways and centrally at the first synapse in the brainstem, where they enhance cough via neurokinin‐1 (NK‐1) receptors (Badri & Smith, 2019; Mazzone et al., 2005). Moreover, NK‐1 receptor antagonists abolish cough responses following microinjection of Substance P into the caudomedial nucleus tractus solitarius (NTS) in animal models (Mazzone et al., 2005). This has led to the investigation of NK‐1 receptor antagonists in RCC; however, these have demonstrated limited clinical utility to date (Birring, Chaudhuri, et al., 2024; Smith et al., 2019; Smith et al., 2020; Smith et al., 2021).

### 4.2 Supramedullary brain regions, voluntary cough and cough reflex inhibition

Humans can exert voluntary control over cough, indicating supramedullary modulation of the reflex (Mazzone et al., 2011). Functional imaging studies reveal distinct cortical networks for reflex cough, voluntary cough and cough suppression (Mazzone et al., 2011; Mazzone et al., 2013; Sugi et al., 2024). Voluntary cough activates the sensorimotor cortex, supplementary motor area and cerebellum (Mazzone et al., 2011; Mazzone et al., 2013; Sugi et al., 2024) whereas reflex cough engages the posterior insula and cingulate cortices to a greater magnitude with minimal brainstem involvement (Mazzone et al., 2011; Sugi et al., 2024). Cough suppression recruits inhibitory regions, including the anterior mid‐cingulate cortex, anterior insula and right inferior frontal gyrus, areas linked to motor inhibition (Mazzone et al., 2011; Mazzone et al., 2013; Sugi et al., 2024). The perception of an ‘urge‐to‐cough’ correlates with widespread cortical activation, modulated by stimulus intensity and overlaps with networks for actual cough (Mazzone et al., 2007). These findings highlight integrated sensory, motor and limbic processing and explain why psychosocial and neurobiological factors, including expectation and voluntary control, drive the substantial placebo response in antitussive trials (Leech et al., 2013; Zhang, Zhang, & Morice, 2024).

Recent studies have highlighted the critical role of the midbrain in cough hypersensitivity (Ando et al., 2016; Moe et al., 2024). It is well established that people with RCC exhibit heightened sensitivity, with cough elicited at lower concentrations of inhaled irritants such as capsaicin and ATP (Ando et al., 2016; Cho et al., 2019; Moe et al., 2024). A recent functional MRI study demonstrated that people with RCC exhibited reduced medullary activity in the nTS and paratrigeminal nucleus compared to healthy controls when exposed to an identical level of capsaicin‐ and ATP‐evoked urge to cough (Moe et al., 2024). Whilst no differences were observed in cortical regions encoding the urge‐to‐cough, greater activity was observed in a region of the midbrain adjacent to the periaqueductal grey (PAG) in RCC compared to healthy controls (Moe et al., 2024). Although the spatial resolution of fMRI prevents the authors from definitively attributing the increased activity seen in this region to the PAG specifically, the PAG is well positioned to influence cough sensory processing and reflex output. This is because the PAG forms part of a descending modulatory network that regulates brainstem reflex circuits, including well‐established roles in pain modulation, respiratory control and defensive behaviours (Makovac et al., 2021; Meier et al., 2017). Collectively, these findings suggest that cough hypersensitivity may arise from central dysregulation, potentially involving altered processing at supramedullary levels, rather than uniform peripheral sensitisation (Fowles et al., 2017; Smith, Chapman, et al., 2025). Whilst this does not negate the therapeutic potential of targeting peripheral nerve sensitivity, it highlights the promise of interventions aimed at restoring central inhibitory function, such as neuromodulation or behavioural therapies. However, given the limitations in spatial resolution and anatomical specificity, further investigation is required to delineate the precise neural substrates involved.

## 5 SUMMARY AND FUTURE DIRECTIONS

The current paradigm of cough pathophysiology comprises cough reflex hypersensitivity and impaired volitional suppression. The former is conventionally considered to be primarily mediated through peripheral nerve hypersensitivity. In turn, to date, efforts have focused on the investigation and antagonism of peripheral afferent receptors, which have yielded mixed results. The lack of consistent clinical translation across receptor targets suggests that the underlying neurophysiology is more complex than can be explained by a single peripheral receptor or pathway. Meanwhile, our current experimental techniques remain largely limited to the interrogation of individual receptors, hence the recent advances in pharmacotherapy developments focusing on peripheral targets. These include modulation of transient receptor channels, such as TRPM8 (e.g. AX‐8) and inhibition of voltage‐gated sodium channels (e.g. NTX‐1175) (Smith et al., 2024; National Library of Medicine (US), 2024). Among emerging therapies, Camlipixant—a selective P2X3 receptor antagonist—has shown particular promise and is currently in phase 3 clinical studies (Smith, Birring, et al., 2025; Smith, Morice, et al., 2025). Concurrently, there is growing recognition of the therapeutic potential of centrally acting agents. Nalbuphine is a centrally acting synthetic opioid with mixed agonist–antagonist properties and has demonstrated encouraging efficacy in recent RCC studies, highlighting the relevance of central neural mechanisms in cough modulation (Smith, Chapman, et al., 2025). Despite these advances, contemporary pharmacological strategies remain largely predicated on single‐target antagonism. Although Nalbuphine is an exception, the broader pharmacodynamic and pharmacokinetic interactions underlying its clinical effects—and, more generally, the complex neurobiology of RCC—remain incompletely understood. In this context, emerging computational approaches, including artificial intelligence and machine learning, may offer transformative insights. By enabling modelling of complex, integrated neural networks, these approaches have the potential to move beyond single‐receptor paradigms and support the development of more comprehensive mechanistically informed therapeutic strategies.

Recent neuroimaging findings further challenge the concept of purely peripherally mediated cough reflex hypersensitivity. Taken together, there is overwhelming evidence to reconsider the current paradigm of cough pathophysiology and pharmacotherapy. Importantly, pharmacotherapy development to date has largely regarded RCC as a homogeneous cohort. Future therapeutic strategies should instead be guided by detailed phenotypic and endotypic characterisation, with a view to targeting multiple peripheral and central pathways. In this context, ligand‐specific antitussive therapies represent a promising avenue, particularly when aligned with individual patient profiles.

Notably, the placebo effect has been prominent in recent P2X2/3 antagonist studies (Birring et al., 2023; McGarvey et al., 2022; McGarvey, Smith, et al., 2023) and remains a persistent challenge in antitussive clinical trials. Moe and colleagues demonstrated reduced neural activity during placebo intervention compared with no intervention in some regions of the medullary brainstem, including the spina trigeminal nucleus, nTs, rostral VRG, cuneiform nucleus, lateral parabrachial nucleus and tegmental nucleus (Moe et al., 2026). These findings may contribute to an improved understanding of the placebo response in RCC and help inform the design of future clinical trials.

## 6 CONCLUSION

In summary, cough hypersensitivity reflects a maladaptive physiological state, involving altered ion channels, heightened vagal signalling and central neuroplasticity. Therapies targeting vagal afferent pathways have shown promise, but variable clinical responses underline the mechanistic heterogeneity of RCC. Integrating mechanistic, translational and ecological approaches will be crucial for identifying robust biomarkers, defining endotypes and developing targeted and effective treatments for RCC.

## AUTHOR CONTRIBUTIONS

**James C. Brown:** Conceptualization; investigation. **Katherine L. Rhatigan:** Conceptualization. **Oliver J. Price:** Conceptualization; supervision. **Peter S. P. Cho:** Conceptualization; methodology; supervision.

## FUNDING INFORMATION

No funding information provided.

## CONFLICT OF INTEREST STATEMENT

JB and KLR have no competing interests. OJP is an Associate Editor at Physiological Reports and was blinded from reviewing or making decisions for this manuscript. Another editor oversaw the manuscript process for this article. OJP has received research funding from Merck, AstraZeneca and the EPSRC outside the submitted work. PSPC reports an investigator‐initiated grant from Merck, consulting fees from GSK and Strados, and an honorarium from GSK and AstraZeneca.

## DISCLOSURE

A narrative literature search was conducted using electronic databases including PubMed, Web of Science, Scopus and Google Scholar. Search terms included combinations of keywords such as ‘refractory chronic cough’, ‘vagal afferents’, ‘cough neural mechanisms’, ‘TRP channels’ and ‘cough reflex hypersensitivity’. Reference lists of selected articles were also screened to identify additional relevant studies. Articles were selected based on their relevance to the neurophysiological mechanisms underlying cough and the pathophysiology of chronic cough. Only peer‐reviewed articles published in English were included. The search primarily focused on literature published between 2000 and 2025, although earlier studies were included where appropriate.

## GUARANTOR STATEMENT

PSPC confirms responsibility for the content of the manuscript on behalf of all authors.
